# Meropenem: continuous or extended infusion?

**DOI:** 10.1186/s13054-020-02883-w

**Published:** 2020-05-05

**Authors:** Frédéric Frippiat, Christelle Vercheval, Nathalie Layios

**Affiliations:** 1 Division of Infectious Diseases, Department of Internal Medicine, University Hospital of Liège, Liège, Belgium; 2 Service de Maladies Infectieuses, CHU de Liège, Avenue de l’hôpital, 1 - B35, Sart Tilman, 4000 Liège, Belgium; 3 Department of Clinical Pharmacy, University Hospital of Liège, Liège, Belgium; 4 Department of Intensive Care Unit, University Hospital of Liège, Liège, Belgium

To the Editor

We read with interest the article by Benitez-Cano and colleagues about intrapulmonary concentrations of meropenem administered by continuous infusion (CI) in critically ill patients with nosocomial pneumonia and would like to make some comments [1].

Firstly, the pharmacokinetic/pharmacodynamic (PK/PD) target was a free epithelial lining fluid (ELF) concentration of 50% of time above MIC (50% *f*T > MIC). In our opinion, a PK/PD target of 100% *f*T > MIC was more suitable, since the study was performed under CI. Indeed, despite the fact that the authors stated that “a precise estimate of the concentration-time profile in ELF was not possible because all ELF samples were obtained at the same time,” CI of β-lactams allows reasonably a 24/24 stable concentration both in plasma and ELF, as illustrated in the figures 2 and 6 for the plasma and ELF, respectively [1], and as shown in other studies [2, 3].

Secondly, considering a target of 50% *f*T > MIC, similar results were obtained with both extended infusion (EI) over 4 h and CI (i.e., MIC up to 1 and up to 2 mg/L for both modes of infusion with 1 g/8 h and 2 g/8 h, respectively), which are close to our results with EI over 3 h (i.e., MICS up to 0.5 and up to 1 mg/L with 1 g/8 h and 2 g/8 h, respectively) [4]. Thus, CI does not offer significant PK/PD advantages over EI for meropenem. On a practical point of view, CI of meropenem needs a dedicated intravenous line access (which is not always obvious in critically ill patients) and frequent infusion syringes changes (every 5–8 h) due to stability issues, particularly at temperatures ≥ 25 °C [5].

Thirdly, studies performed in critically ill patients with nosocomial pneumonia showed a high interindividual variability in the β-lactams concentrations in ELF whatever the mode of infusion [1–4]. We agree with Benitez-Cano et al. that even the highest dosage of meropenem (2 g/8 h) administered by either CI or EI could not result in an optimal ELF target attainment for a substantial fraction of the population, particularly in patients with augmented renal clearance.

In conclusion, when meropenem is considered as the initial empiric antibiotic therapy for nosocomial pneumonia in critically ill patients, we strongly recommend the dosage of 2 g/8 h by EI over 3 h (or by CI if the cartridge is changed every 5–8 h and the temperature remains below 25 °C) to optimize chances for therapeutic concentrations in ELF.

## Data Availability

Not applicable
